# Low socioeconomic position is a risk factor for delay to treatment and mortality of testicular cancer patients in Hungary, a prospective study

**DOI:** 10.1186/s12889-021-11720-w

**Published:** 2021-09-19

**Authors:** Zsófia Küronya, Georgina Fröhlich, Andrea Ladányi, Tamás Martin, Lajos Géczi, Fruzsina Gyergyai, Orsolya Horváth, Gergő Kiszner, Ágnes Kovács, Tamás Dienes, Enikő Lénárt, Krisztián Nagyiványi, Tibor Szarvas, Mihály Szőnyi, Attila Tóth, Krisztina Biró

**Affiliations:** 1 Department of Genitourinary Medical Oncology and Clinical Pharmacology, National Institute of Oncology, Ráth György u. 7-9, Budapest, H-1122 Hungary; 2 Faculty of Science, Eötvös Loránd University, Budapest, Hungary; 3 Centre of Radiotherapy, National Institute of Oncology, Budapest, Hungary; 4 Department of Surgical and Molecular Pathology, National Institute of Oncology, Budapest, Hungary; 5 Boehringer Ingelheim RCV GmbH & Co. KG Branch Office Budapest, Budapest, Hungary; 6 Department of Urology, Semmelweis University Budapest, Budapest, Hungary; 7 Semmelweis University Faculty of Medicine, Budapest, Hungary; 8 Adherencia.net Kft., Budapest, Hungary

**Keywords:** Testicular cancer, Socioeconomic position, Patient delay

## Abstract

**Background:**

In Hungary, the mortality rate for testicular germ cell cancer (TGCC) is 0,9/100000 which is significantly higher than the EU average. We prospectively evaluated the effect of socioeconomic position on patient delay and therapy outcomes.

**Methods:**

Questionnaires on subjective social status (MacArthur Subjective Status Scale), objective socioeconomic position (wealth, education, and housing data), and on patient’s delay were completed by newly diagnosed TGCC patients.

**Results:**

Patients belonged to a relatively high socioeconomic class, a university degree was double the Hungarian average, Cancer-specific mortality in the highest social quartile was 1.56% while in the lowest social quartile 13.09% (*p* = 0.02). In terms of patient delay, 57.2% of deceased patients waited more than a year before seeking help, while this number for the surviving patients was 8.0% (*p* = 0.0000). Longer patient delay was associated with a more advanced stage in non-seminoma but not in seminoma, the correlation coefficient for non-seminoma was 0.321 (*p* < 0.001). For patient delay, the most important variables were the mother’s and patient’s education levels (r = − 0.21, *p* = 0.0003, and r = − 0.20, *p* = 0.0005), respectively. Since the patient delay was correlated with the social quartile and resulted in a more advanced stage in non-seminoma, the lower social quartile resulted in higher mortality in non-seminoma patients (*p* = 0.005) but not in seminoma patients (*p* = 0.36) where the patient delay was not associated with a more advanced stage.

**Conclusions:**

Based on our result, we conclude that to improve survival, we should promote testicular cancer awareness, especially among the most deprived populations, and their health care providers.

**Supplementary Information:**

The online version contains supplementary material available at 10.1186/s12889-021-11720-w.

## Background

Malignant testicular germ cell cancer (TGCC) is relatively uncommon, however, this is still the leading type of cancer in men between the ages of 20 and 40 years [1]. Due to huge improvements in imaging and chemotherapy, mortality rates in patients with testicular cancer have declined in recent decades. In the most affluent world regions, while rapid increases in incidence rates have been observed lately, the mortality rates declined to 0.2–0.3 per 100,000. However, in Central and Eastern European (CEE) countries (i.e., Bulgaria, Czech Republic, Hungary, Poland, Romania, and Slovakia), rates have only moderately declined and were still 1.3/100,000 [2–4] for men aged between 20 and 44 years in 1995–1997. In the first decade of the 2000s, a significant decline in testicular cancer patients’ mortality rates appeared also in CEE countries, but the ratio is still higher than in the more developed EU members [5]. According to a WHO database, Hungary and Latvia had the worst TGCC mortality rate in Europe (0.9/100,000) between 2000 and 2006 [6]. The slower and delayed declines in the less developed, lower resource countries imply that the high cost of appropriate treatments together with inadequate patient referral systems are responsible for the high mortality rates and less favourable trends. Another factor of the slower progress may partly relate to differences in socioeconomic position (SEP), i.e., men with lower SEP may be characterized by a lack of awareness and may be less likely to seek immediate medical help, moreover, they tend to have the worst access to medical service, particularly in remote and rural communities. There is extensive literature describing SEP differences and cancer survival in countries around the world in various health care systems [7–12]. The authors of this report prospectively evaluated the effects of socioeconomic position on delay to diagnosis, stage distribution, and cancer-specific mortality (CSM) in men with TGCC. The studied patient cohort was treated at the National Institute of Oncology GU department, which is the main germ cell cancer center in Hungary, treating roughly 60% of the approximately 600 Hungarian TGCC patients yearly. Diagnosis of cancer can make it difficult to measure the socioeconomic position (SEP), since patients may lose their regular income during treatment, and asking about it may become a particularly sensitive issue. Due to this difficulty, several studies suggest using subjective social status (MacArthur Subjective Status Scale-SSS) instead of traditional SEP [13–17]. Since SSS is easier to measure the current study examined how SSS correlates with SEP indicators among Hungarian testicular cancer patients.

The aim of this research was to investigate how the patients’ socioeconomic position affects the time elapsed between onset of symptoms and diagnosis, and therapy outcome, and how objective socioeconomic position correlates with subjective social status among testicular cancer patients.

## Methods

### Ethical statement

This prospective, non-interventional study was carried out between January 2016 and January 2018, at the National Institute of Oncology. The database lock occurred in September 2020. The study was performed in accordance with Hungarian laws and regulations and was approved by the Hungarian National Scientific Ethical Committee (approval number: 44476–2/2016), and as per the standards of the World Medical Association Declaration of Helsinki. All patients who were willing to participate signed informed consent.

### Study population

Inclusion criteria included male patients over 16 years old (inclusive), with a gonadal onset germinal cancer. Every new patient completed the questionnaire on their first visit to our department.

Clinical stage and prognosis were determined according to the Union for International Cancer Control (UICC) [18], and the International Germ Cell Cancer Collaborative Group (IGCCCG) [19] based on the pathology report, the tumor marker (AFP, HCG, LDH) level, and the CT scan. Castration was the first-line treatment for the majority of patients (Table 1), followed by surveillance or adjuvant chemotherapy (St I), or by multiple cycles of platinum-based chemotherapy, and salvage surgery where needed (St II-III). In 16 patients whose conditions related to metastatic dissemination required immediate chemotherapy, orchiectomy was postponed until the completion of chemotherapy. Patients received the internationally recognized standard of care, and after completion of treatment were followed based on the current European Society for Medical Oncology (ESMO) guideline [20]. The median observation time is 35 months. During this period no patient was lost to follow up.

**Table 1:** Characteristics of the surviving and the deceased patients

	Surviving patients (*N* = 289)	Deceased patients (*N* = 14)	
	mean ± SD		
Age of patient	35.9 ± 9.8	41.62 ± 12.3	*p = 0.0277
Age of mother at childbirth	26.1 ± 5.5	24.92 ± 6.4	*p = 0.4397
Age of father at childbirth	29.4 ± 6.5	27.77 ± 7.8	*p = 0.4186
Histology	N		
Seminoma	151 (98.1%)	3 (1.9%)	
Non-seminoma	138 (92.6%)	11 (7.4%)	**p = 0.0289
First-line treatment	N		
Castration	277 (95.8%)	10 (71.4%)	**p = 0.0038
Neo-adjuvant chemotherapy followed by castration	12 (4.2%)	4 (28.6%)	
Education	N		
Primary school or below	21 (7.3%)	2 (14.3%)	**p = 0.0012
Vocational school	65 (22.7%)	10 (71.4%)	
High School	98 (34.1%)	0 (0.0)	
University/college	103 (35.9%)	2 (14.3%)	
Settlement	N		
Budapest	94 (32.5%)	1 (7.1%)	**p = 0.0271
County seat	38 (13.1%)	2 (14.3%)	
City	97 (33.6%)	5 (35.7%)	
Village	60 (20.8%)	6 (42.9%)	
Prognosis	N		
Good	262 (90.8%)	5 (35.7%)	**p < 0.001
Intermediate	12 (4.3%)	2 (14.3%)	
Poor	14 (4.9%)	7 (50.0%)	
Patient’s delay	N		
≤ 1 week	109 (37.7%)	1 (7.1%)	****p < 0.001
1 week–1 month	102 (35.2%)	1 (7.1%)	
1–6 months	45 (15.6%)	3 (21.5%)	
6–12 months	10 (3.5%)	1 (7.1%)	
> 12 months	23 (8.0%)	8 (57.2%)	
Social quartile (SES)	N		
1	73 (25.3%)	11 (78.7%)	**p < 0.001
2	66 (22.8%)	1 (7.1%)	
3	62 (21.4%)	1 (7.1%)	
4	63 (21.8%)	1 (7.1%)	
N.D.	25 (8.7%)	0 (0.0%)	

N: number of patients, SD: standard deviation, N.D. No data, *Student’s t-test, **Fisher-exact test, ***Mann-Whitney U test, ****logistic regression

### Assessment of the subjective social status and objective socioeconomic position

**Fig. 1:**
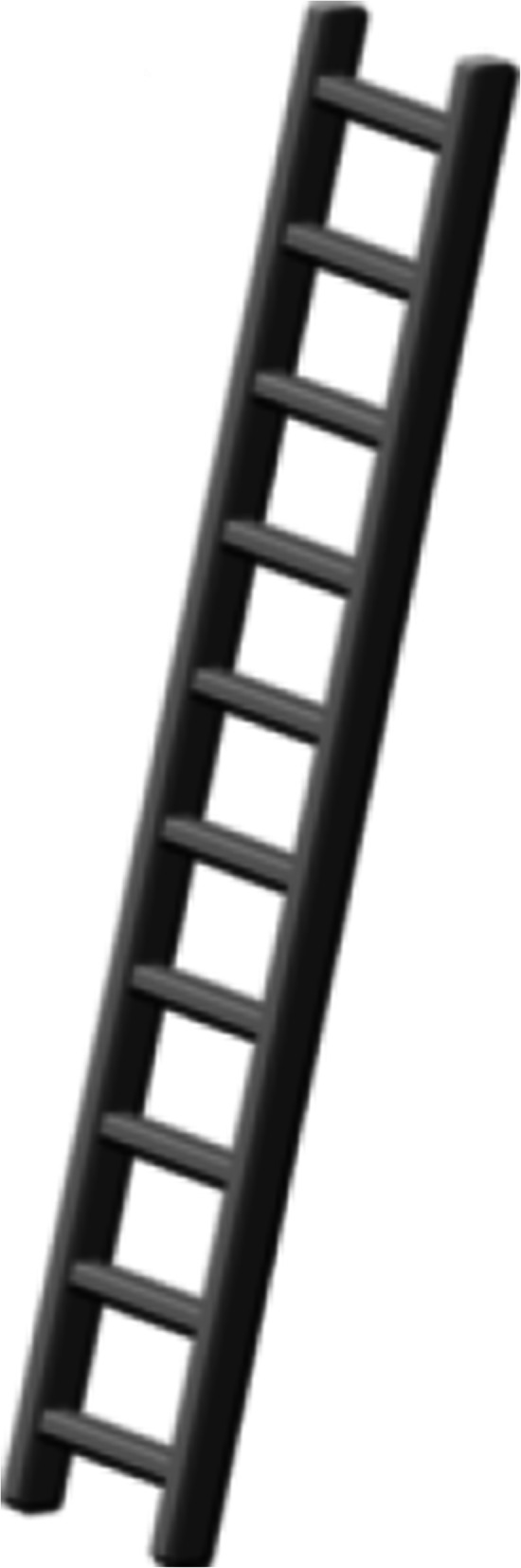
Illustration of the ladder used in the “MacArthur” Scale of Subjective Social Status Retrieved from www.macses.ucsf.edu [21] (used with the author’s written permission) Social status value: At the top of the ladder are the people who are the best off, those who have the most money, the most education, and the best jobs. At the bottom are the people who are the worst off, those who have the least money, least education, worst jobs, or no job Community status value: Think of this ladder as representing where people stand in their communities. People define community in different ways; please define it in whatever way is most meaningful to you. At the top of the ladder are people who have the highest standing in their community. At the bottom are the people who have the lowest standing in their community “Please place an ‘X’ on the rung that best represents where you think you stand on the ladder”

The subjective social status (SSS) and objective socioeconomic position (SEP) questionnaires were completed on the first consultation visit of newly diagnosed TGCC patients. The SSS was determined based on the MacArthur Scale of Subjective Status questionnaire [21]. After the owner’s written permission for usage and linguistic validation, the questionnaire has been translated into Hungarian and was adapted for this study. MacArthur scale is a social or community hierarchy ladder used by the patients based on their deemed place; with values of 1–10 [13, 16, 22, 23]. We measured two variables: social status, and community status value (Fig. 1). For objective SEP patient’s highest educational attainment, residence type, size of dwelling (m^2^ floor area), the number of household members, number of members with income, internet usage frequency possession of consumer durable goods, and the parents’ highest educational level were evaluated. (Table 3). The SEP questionnaire was developed especially for this study, and you can find the English version of it as a supplementary file (supplementary file). We calculated an index depending on the patient’s highest educational level and wealth. Based on this index, patients were divided into 4 quartiles (SEP quartiles). Since income is a sensitive piece of data, wealth was calculated indirectly based on the number of consumer durable goods and the calculated size of living area per capita. For the patient highest educational level, see Table 3. The first quartile was the lowest, with the poorest status and lowest educational level. The section on illness comprised of questions including the time gap between the first symptoms and visiting a doctor (patient delay, PD). Permanent address, histology, stage, and treatment outcome were extracted from the patient database.

### Statistical analyses

Statistical analyses were performed using SPSS 22.0 (IBM Corp.) and Statistica 12.5 (StatSoft, Tulsa, OK, USA). The correlation between the patient’s delay and survival was determined by logistic regression. To examine the effect of SEP on survival Fisher exact test was carried out. The association between various patient characteristics and OS was analyzed by Kaplan-Meier analysis. To examine the correlation between patient education and PD, and between SSS and SEP, Spearman rank correlation was performed. Results were considered significant at *p* values lower than 0.05.

## Results

### Patient characteristics

Altogether, 306 patients filled out the questionnaire. Analyses were performed on 303 patients who met the inclusion criteria (3 cases with non-gonadal origin were excluded from the analysis). The patients’ mean age was 35.9 years (seminoma 38.5, non-seminoma 33.1) (Table 2). The mother’s mean age at childbirth was 26 years whilst the father’s mean age at childbirth was 29.1 years. Patients belonged to a relatively high socioeconomic class, 103 (34%) patients had a university or college degree, which is twice as many as the Hungarian society average [24]. SSS among patients was 5.57 ± 1.70 while the Hungarian average among males in this age group is 3.97 ± 0.03 (out of 10) [25]. Fourteen patients died during the study period, all death was related to testicular cancer, hence OS and CSS were identical. Deceased patients were significantly older than the surviving patients, 41.6 vs. 35.9 years (*p* = 0.0277) (Table 1). Among patients with metastatic seminoma 152 (98.7%) belonged to the good and 2 (1.3%) to the intermediate prognostic group, while among those with non-seminoma 116 (77.9%), 12 (8.0%), and 21 (14.1%) belonged to good, intermediate, and poor prognostic group, respectively. Table 2 shows the stage and prognosis distribution among seminoma and non-seminoma patients. The majority of patients belonged to stage I (seminoma: 71.5% and non-seminoma 47.6%), though a higher proportion of non-seminoma patients had metastatic disease (StII/StIII) at diagnosis (28.5% seminoma vs. 52.4% non-seminoma).

**Table 2:** Patient characteristics

	Seminoma (*N* = 154)	Non-seminoma (*N* = 149)
Age	mean ± SD (range)	mean ± SD (range)
Patient age (years)	38.5 ± 8.8 (22–64)	33.1 ± 9.6 (17–77)
Mother’s age at childbirth (years)	25.8 ± 5.2 (14–44)	26.2 ± 5.7 (16–43)
Father’s age at childbirth (years)	28.9 ± 6.1 (16–58)	29.3 ± 6.6 (17–54)
Socioeconomic position		
School type – Patient’s education	N	
Primary school or below	6 (3.9%)	17 (11.4%)
Vocational school	37 (24.0%)	38 (25.5%)
High school	53 (34.4%)	47 (31.5%)
University/college	57 (37.0%)	46 (30.9%)
N.D.	1 (0.7%)	1 (0.7%)
School type – Mother’s education	N	
Primary school or below	26 (16.9%)	26 (17.4%)
Vocational school	43 (27.9%)	37 (24.8%)
High school	51 (33.1%)	52 (34.9%)
University/college	31 (20.1%)	29 (19.5%)
N.D.	3 (2.0%)	5 (3.4%)
School type – Father’s education	N	
Primary school or below	12 (7.8%)	24 (16.1%)
Vocational school	64 (41.6%)	54 (36.3%)
High school	45 (29.2%)	27 (18.1%)
University/college	28 (18.2%)	34 (22.8%)
N.D.	5 (3.2%)	10 (6.7%)
Patient’s residence type	N	
Budapest (capital)	49 (31.8%)	48 (32.2%)
County seat	28 (18.2%)	15 (10.1%)
City	48 (31.2%)	55 (36.9%)
Small town/village	29 (18.8%)	31 (20.8%)
Distance from the center – km (N.I.O.)	Mean (range)	
	80.3 (10–300)	87.3 (10–300)
Number of household members	Mean (range)	
	3.0 (1–8)	3.0 (1–7)
Number of members with income	Mean (range)	
	2.0 (0–6)	2.0 (1–5)
Internet usage frequency	N	
Daily	127 (82.5%)	130 (87.2%)
Few times a week to a few times a month	19 (12.3%)	14 (9.4%)
Less frequently	8 (5.2%)	5 (3.4%)
Stage	N	
IA	101 (65.6%)	46 (30.9%)
IB	9 (5.9%)	23 (15.4%)
IS	0 (0.0%)	2 (1.3%)
IIA	15 (9.7%)	19 (12.8%)
IIB	10 (6.5%)	15 (10.1%)
IIC	15 (9.7%)	7 (4.7%)
IIIA	2 (1.3%)	9 (6.0%)
IIIB	2 (1.3%)	13 (8.7%)
IIIC	NA	15 (10.1%)
Prognosis	N	
Good	152 (98.7%)	116 (77.9%)
Intermediate	2 (1.3%)	12 (8%)
Poor	N.A	21 (14.1%)

Separated based on histology (seminoma, non-seminoma). N: number of patients, SE: standard error, N.I.O.: National Institute of Oncology, N.D.: no data, N.A. not applicable

NA: not applicable

### Objective socioeconomic position and subjective social status

Regarding patients’ residence type, distance from the National Institute of Oncology, number of household members, number of members with income, and internet usage frequency there was no difference between the seminoma and non-seminoma groups (Table 2). Table 3 presents social quartiles based on objective socioeconomic position and the distribution of SSS. For SSS the full range of scores (1–10) occurred and their distribution was normal. (Table 3). The mean SSS value for the entire sample was 5.57 ± 1.70. In terms of SSS, there was also no difference between seminoma and non-seminoma patients (*p* = 0.7898).

**Table 3:** Socioeconomic position (SEP) and subjective (SSS) social status value

Social quartile (SES)	Seminoma (*N* = 154)	Non-seminoma (*N* = 149)
1	39 (25.3%)	45 (30.2%)
2	40 (26.0%)	27 (18.1%)
3	33 (21.4%)	30 (20.1%)
4	31 (20.2%)	33 (22.2%)
N.D.	11 (7.1%)	14 (9.4%)
Social status value (SSS)	N	
1	2 (1.3%)	2 (1.3%)
2	4 (2.6%)	4 (2.7%)
3	12 (7.8%)	8 (5.4%)
4	19 (12.3%)	20 (13.4%)
5	31 (20.1%)	38 (25.5%)
6	36 (23.4%)	33 (22.2%)
7	26 (16.9%)	23 (15.4%)
8	15 (9.7%)	16 (10.7%)
9	4 (2.6%)	4 (2.7%)
10	2 (1.3%)	0 (0.0%)
N.D.	3 (2.0%)	1 (0.7%)

N.D.: no data

### Patients’ objective socioeconomic position versus subjective self-grade values

The patient’s subjective social status values were compared to the social quartiles. Both the social ladder value and the community ladder value exhibited a significant correlation with the objective socioeconomic position-based quartiles (r = 0.508 and r = 0.417, respectively, *p* < 0.001).

### Factors associated with patient’s delay

The diagnostic time path was from within 1 week to over 1 year, with the ‘1 week-1 month’ being the median (102 patients). PD negatively correlated with social quartile (r = − 0.18, *p* = 0.0022). We also checked the effect of the elements of SEP separately, including those that were not part of the social quartile. (Table 3). We found a negative correlation between the father’s education and patient delay (PD) (r = − 0.12, *p* = 0.0383), and an even stronger negative correlation between the mother’s education and the PD, as well as the patient’s education and the PD (r = − 0.21, *p* = 0.0003, and r = − 0.20, *p* = 0.0005, respectively). Table 4 shows PD based on the educational level of the patients. The remaining factors showed no correlation with PD. PD was significantly longer for deceased patients than for surviving patients (Table 1).

**Table 4:** Patient’s delay (first visit to GP) based on educational level – all patients

Patient delay (time)	Primary school or below (*N* = 23)	Vocational school (*N* = 75)	High school (*N* = 100)	University/ college (*N* = 103)
≤1 week	7 (30.4%)	19 (25.3%)	38 (38.0%)	46 (44.6%)
1 week–1 month	6 (26.1%)	25 (33.3%)	33 (33.0%)	38 (36.9%)
1–6 months	3 (13.1%)	14 (18.6%)	19 (19.0%)	11 (10.7%)
6–12 months	1 (4.3%)	2 (2.6%)	4 (4.0%)	3 (2.9%)
> 12 months	6 (26.1%)	14 (18.6%)	6 (6.0%)	5 (4.9%)
N.D.		1 (1.3%)		

Spearman rank correlation: −0.2, *p* = 0.0005

### Patient delay, social quartile, and overall survival

**Fig. 2:**
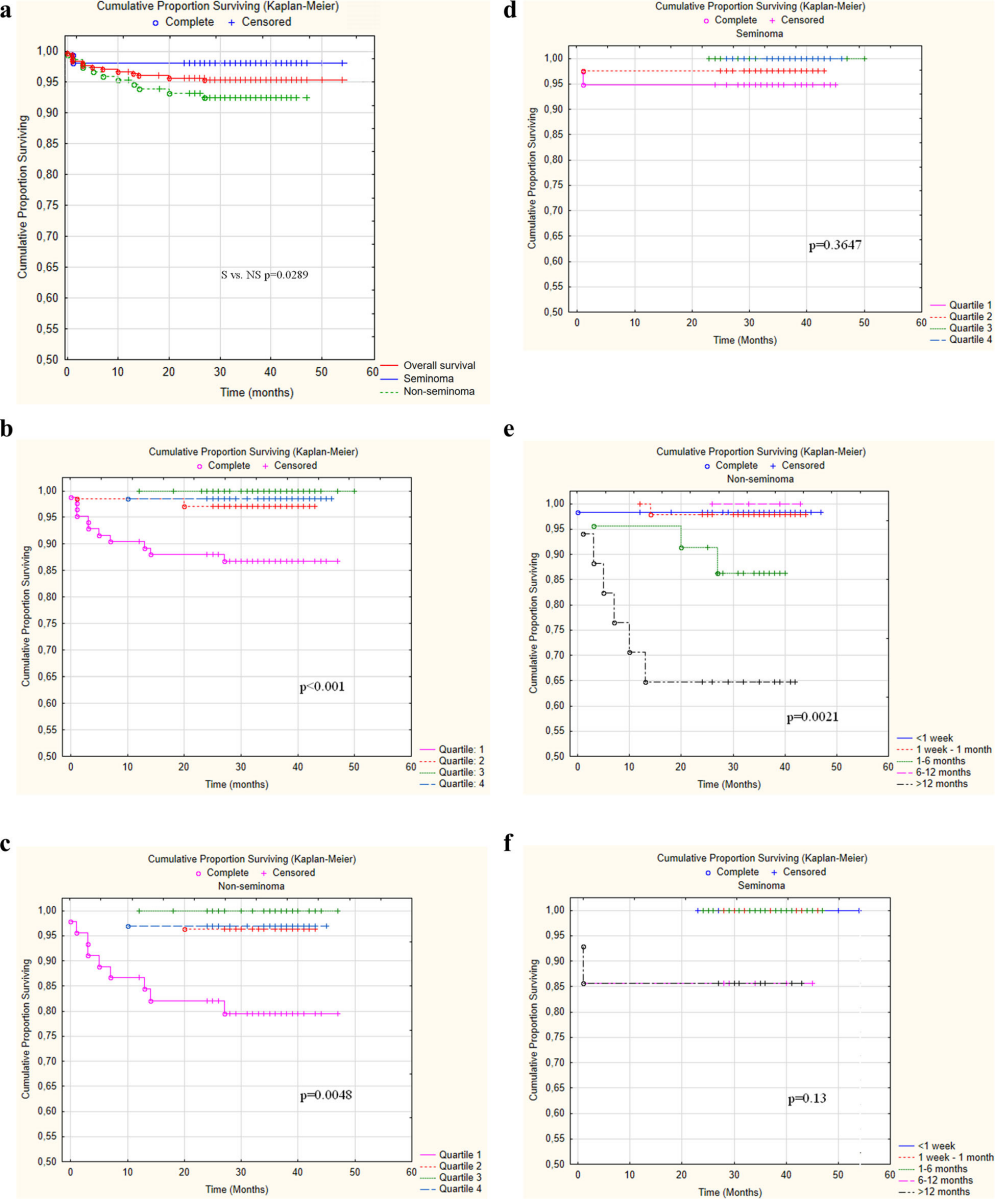
Kaplan-Meier curves for all patients vs. seminoma vs. non-seminoma patients (**a**), patients separated according to social quartile: all patients, (**b**) non-seminoma (**c**), seminoma, (**d**) patient delay: non-seminoma (**e**), seminoma (**f**)

Of the 14 patients who died during the study course, 11 (78.6%) were in the social quartile 1 (lowest), compared to 73 of the 289 surviving patients (25.3%) (*p* < 0.001) (Table 4) Fig. 2 shows the OS based on the social quartile, and PD for all patients and separated for non-seminoma and seminoma patients. 57.2% of deceased patients waited more than a year before seeking help, while this number for the surviving patients was 8.0% (p < 0.001). Longer PD was associated with a more advanced stage in non-seminoma, but not in seminoma patients, the correlation coefficient for NS was 0.321 (p < 0.001), for S (*p* = 0,081), hence PD significantly influenced OS in NS (*p* = 0.0021) but not in S (*p* = 0.13) (Fig. 2). Since PD was correlated with the social quartile, as mentioned above, and resulted in a more advanced stage in non-seminoma, a lower social quartile resulted in higher mortality in NS patients (*p* = 0.0048) but not in S patients (*p* = 0.36) where PD was not associated with more advanced stage.

## Discussion

The present study has been conducted at one site (National Institute of Oncology, Department of Genitourinary Medical Oncology and Clinical Pharmacology), between 2016 and 2018, and includes the prospective data analysis of the socioeconomic position of patients treated with testicular cancer, or in case of early-stage followed without treatment (surveillance). The 303 patients who participated in the study, are approximately half of the yearly Hungarian TGCC incidence (600–650 cases/year) [26].

Inequalities in cancer mortality and morbidity between populations with different (lower and higher) socioeconomic positions are widely described in the literature [27]. We measured both the SSS (social ladder) and SEP and found that both the social ladder value and the community ladder value exhibited a significant correlation with the objective socioeconomic position-based quartiles. The association between SEP and SSS, which is distinctive in different cultural groups, was reported in several papers [28, 14, 29]. SSS is easier to use because many people do not want to report their income and education levels. Furthermore, it seems to predict health and general well-being better than objective SEP measurements [23, 30].

In the current study, we found a major deviation in terms of the patient’s highest education level compared to the country averages Those educated to college/university level (34%) were represented 1.5 times more as the Hungarian society averages suggest, while those with the lowest education (8%) have been presented in a significantly lower extent compared to the Hungarian average (27%) [24]. SSS among patients was 5.57 ± 1.70 while the Hungarian average among males in this age group is 3.97 ± 0.03 (out of 10) [25]. Several studies found that testicular cancer occurs more frequently among men of high SEP, and among sons of mothers of high SEP [31, 32], while other reports have not found such associations [33]. A more recent report suggests that the differences have decreased in recent years. In the USA between 1973 and 2008, the TGCC incidence rate for persons with low SEP was lower than the rate for those with high SEP but increased at a faster rate, and the two incidence rates got close to each other by 2008 (5.8/100,000 for low SEP and 6.2/100,000 for high SEP) [34]. Since both the intensity of treatment and prognosis are based on the extent of disease at presentation, a testicular cancer diagnosis must not be delayed. Among our TGCC patients, PD showed significant relations with SEP. In terms of PD, the most influential was the mother’s and patient’s education. Delay in the diagnosis of testicular cancer is well documented [35–38]. In Dieckmann and colleague’s paper PD was found to be related to educational level, i.e. college-educated men had a shorter median delay [38], while Post and Bellis found that the less educated men tend to connect the larger size of the testicle with more virility [39]. On the other hand, Toklu et al. have not detected a correlation between the annual income, the educational level, and the delay to treatment [40]. There is only a limited reference in the literature to the possible association between the patient’s SEP and the stage of their disease. SEER data from 2011 showed that patients living in high poverty areas and those with lower educational levels have a higher chance of an advanced TGCC diagnosis [34]. Seminomas can have indolent growth, and delay in diagnosis usually does not result in a more advanced stage [38, 39, 41]. Since in our study PD in seminoma patients did not influence the stage, thus the social quartile in seminoma did not determine the survival significantly. In the case of non-seminoma, the connection between delay in diagnosis and advanced disease is more straightforward [36] [37, 42, 43], and hence longer delay has resulted in decreased survival [39, 41, 44]. PD among our non-seminoma patients presented a more advanced stage, and based on our research, PD was significantly correlated with SEP, therefore social quartile and survival also had significant interdependence in non-seminoma patients. Although Marså et al., found no substantial social gradients in the incidence of or survival from testicular cancer in Denmark [45], in the study of Davies, TGCC patients with lower SEP had higher mortality rates [46]. Sun et al. reported from their multivariate analysis that low SEP groups had significantly higher cancer-related mortality rates, as well as higher collective mortality rates retrospectively [47]. This was in line with the findings of Davies et al. who reported a higher mortality rate among those educated at the vocational school level [46]. This has also been proved by the results reported in this research, as 10 out of 14 deceased patients were educated to that level, and 9 out of 10 belonged to the lowest quartile. The number of deceased patients was significantly higher among those who waited at least 1 year before the first doctor visit. According to our knowledge, there is no similar finding, published in our research area.

The survival rate among our patients (95.4%) was better compared to the Hungarian average (91–93%) [26, 48], and this underscores the importance of having the TGCC patients managed in a specialized center. It has been reported earlier that specialist centers demonstrate superior results to nonspecialist centers [49, 50].

### Limitation of the study

Similar to other studies measuring patient-reported timelines, bias may occur when patients recall the exact delay period.

## Conclusion

To our knowledge, this is the first study that prospectively evaluated the TGCC patients’ subjective social status and objective socioeconomic position along with survival, all other studies investigated them retrospectively, and indirectly. Mother’s and patient education posed an influent aspect of PD; higher education led to a shorter PD period and hence better survival in non-seminoma patients.

Although early detection is key to better survival, with lower morbidity, delay in diagnosis in Hungary is still a serious problem. Both patients and physicians may contribute to this issue. Patient procrastination derives from ignorance, fear of cancer diagnosis, embarrassment, or fear of losing masculinity. Currently, the U.S. Preventive Services Task Force doesn’t recommend mass screening for testicular cancer among asymptomatic males, but the American Cancer Society suggests testicular inspection as part of a male physical exam, for early detection. Meanwhile, physicians should also promote testicular self-examination, since testicular cancer awareness is low among young adults, which in turn contributes to diagnostic delays, especially among the most underprivileged communities. Based on our results, in Hungary, we should educate young men about the symptoms of testicular cancer and encourage them to turn to their physician at the first sign of a testicular lump, or other pathological changes. Besides, we must continue to educate health care providers, especially primary care physicians. On the other hand, it would be a good idea to incorporate the subject in the secondary school curriculum, as part of health education.

## Supplementary Information

**Additional file 1.** SEP questionnaire (English version).

## Data Availability

The datasets used and/or analysed during the current study are available from the corresponding author on reasonable request.

## Abbreviations

AFP: Alpha-fetoprotein
CEE: Central and Eastern European countries
CT: Computed tomography
CSM: Cancer-specific mortality
EU: European Union
GU: Genito-urinary
HCG: Human chorionic gonadotropin
IGCCCG: International Germ Cell Cancer Collaborative Group
Km: Kilometer
LDH: Lactate dehydrogenase
OS: Overall survival
PD: Patient delay
SEP: Socioeconomic position
SSS: Subjective social status
St: Stage
TGCC: Testicular germ cell cancer
UICC: Union for International Cancer Control
WHO: World health organization
