# Supplementary material for: Low socioeconomic position is a risk factor for delay to treatment and mortality of testicular cancer patients in Hungary, a prospective study
Source: BMC Public Health. 2021 Sep 19;21:1707. doi: 10.1186/s12889-021-11720-w (PMC8451119; doi:10.1186/s12889-021-11720-w)
Supplement: Supplementary file 1 — Additional file 1. SEP questionnaire (English version). [file 12889_2021_11720_MOESM1_ESM.docx]

**Supplement:**

**1. Your date of birth:** ______

**2- Your mother’s date of birth:** ______

**3. Your father’s date of birth:** ______

**4. What is the highest level of education (grade or year) you have completed?** **:**

1. 8th grade or less
2. Vocational school
3. High school
4. University/college

**5. If you are still studiing what is your goal in education?**

1. 8th grade or less
2. Vocational school
3. High school
4. University/college

**6. What is the highest level of education (grade or year) your mother has completed?**

1. 8th grade or less
2. Vocational school
3. High school
4. University/college

**7. What is the highest level of education (grade or year) your father has completed?**

1. 8th grade or less
2. Vocational school
3. High school
4. University/college

**8. How many people are currently living in your household, including yourself?**

______

**9. How many of them have regular income ?**

______

**10. What is the total living space of the flat/ apartment/house you live in (with others)**

______ squer meters

**11. What type of settlement do you live in?**

1. Capital city- Budapest
2. County seat
3. City
4. Small town/village

**12. Which county do you live in?**

| 01. Budapest | 11. Jász-Nagykun-Szolnok |
| --- | --- |
| 02. Bács-Kiskun | 12. Komárom-Esztergom |
| 03. Baranya | 13. Nógrád |
| 04. Békés | 14. Pest |
| 05. Borsod-Abaúj-Zemplén | 15. Somogy |
| 06. Csongrád | 16. Szabolcs-Szatmár-Bereg |
| 07. Fejér | 17. Tolna |
| 08. Győr-Moson-Sopron | 18. Vas |
| 09. Hajdú-Bihar | 19. Veszprém |
| 10. Heves | 20. Zala |

**13. Which of the following consumer durable goods do you have in your household? You can choose more than one answer/item**

1. Dishwasher
2. LCD / PlasmaTV
3. PC (personal computer)
4. Laptop
5. Tablet
6. Subscripton-based cell phone (mobil phone)
7. Prepaid mobile phone (cell phone)
8. Smart phone
9. Cable internet access
10. Mobile internet access
11. Car over 6 years old
12. Car below 6 years old

**14. Community comparison of social standing**

Think of this ladder as representing where people stand in your community. People

define community in different ways; please define it in whatever way is most meaningful

to you. Imagine everyone in your community is standing somewhere on this ladder. At

the TOP of the ladder are the people who have the highest standing in your community.

At the bottom are the people who have the lowest standing in your community. The

higher up you are on this ladder, the closer you are to those at the very top. The lower

you are, the closer you are to those at the very bottom.

**Where would you place yourself on this ladder, compared to others in your**

**community? Please place a large “X” directly on the rung where you think you stand.**

**15. Hungary comparison of socio-economic status.**

Think of this ladder as representing where the people stand in Hungary. At the

TOP of the ladder are the people who are the best off—those who have the most money,

the most education, and the most respected jobs. At the BOTTOM are the people who

are the worst off—who have the least money, least education, and the least respected jobs

or no job. The higher up you are on this ladder, the closer you are to the people at the

very top. The lower you are, the closer you are to the people at the very bottom.

Where would you place yourself on this ladder, compared to all the other people in

Hungary? Please place a large “X” directly on the rung where you think you stand

**16.** Think of this ladder as representing where the people stand in your workplace – different staff hold different recognised position. If you are a student, this ladder represents your school/class; where think of your position. TOP of the ladder (10 point) are the people who are the most recognized, best appreciated – the BOTTOM ( 1 point) who are the less appreciated. Please place a large “X” directly on the rung where you think you stand.

**17. How often do you use the internet?**

1. Each day
2. Several times per week
3. Weekly
4. Several times per month
5. Less often

**18. What do you use the internet for? You can choose more than 1 answer.**

1. Emailing
2. Reading news
3. Reading others’ opinion; forums
4. Facebook
5. Skype / MSN / etc
6. Collecting information; searching website
7. Sharing photos and videos
8. Managing administrative affairs
9. Shopping
10. Playing
11. Blog wrinting/reading
12. Banking
13. Movie, music
14. Other, please specify it___________________

**19. When did you visit your doctor after your symptoms appeared?**

1. Within 1 week
2. Within 1 month
3. Within 6 months
4. Within one year
5. After over a year
